# Recovery Mentors as continuing professional development trainers for better recognition of the epistemic value of the experiential knowledge and improved access to recovery-oriented practices

**DOI:** 10.3934/publichealth.2019.4.447

**Published:** 2019-10-25

**Authors:** Jean-François Pelletier, Larry Davidson, David Gaulin, Jonathan Bordet

**Affiliations:** 1 Research Centre of Montreal Mental Health University Institute, Montreal East Island Integrated University Health and Social Services Centre, Montreal QC, Canada; 2 Program for Recovery & Community Health, Department of Psychiatry, Yale University, New Haven CT, USA; 3 Department of Psychiatry & Addictology, University of Montreal, Montreal QC, Canada; 4 Department of Mental Health and Addictions, Montreal South-Center Island Integrated University Health and Social Services Centre, Montreal QC, Canada

**Keywords:** Recovery Mentors, peer support workers, epistemic injustice, Actor Network Theory, continuing professional development, mental health

## Abstract

**Objectives:**

To lay the groundwork for the arrival of Recovery Mentors (RMs) in some of its multidisciplinary teams, a Continuing Professional Development (CPD) conference was organized in a large public agency in the province of Quebec, Canada. The aim was to come up collectively with recommendations to improve access to recovery-oriented care and services for this vulnerable population by recognizing the epistemic value of their lived experience.

**Methods:**

A series of workshops were organized among health professionals to reflect on their practice and to discuss the role of RMs for improving epistemic equity and recognition of the experiential knowledge. In preparation for these workshops participants completed the Recovery Self-Assessment (RSA). The RSA is a 32-item questionnaire designed to gauge the degree to which programs implement recovery-oriented practices, which should notably include RMs in multidisciplinary teams (five-point Likert scale: 1= *strongly disagree* ; 5 = *strongly agree*). The interactive workshops were hosted by RMs as trainers who first shared their lived experience and understanding of recovery.

**Results:**

Eighty-height of the 105 participants completed the RSA. The highest score on the RSA was for the item *Staff believe in the ability of program participants to recover* (mean = 4.2/5). The lowest score was for the item *People in recovery are encouraged to attend agency advisory boards and management meetings* (mean = 2.2/5). Based on the average inter-item correlation, a reliability test confirmed an excellent internal consistency for the French RSA scale, with a Cronbach's Alpha of .9. Means and standard deviation for each item of the RSA questionnaires were calculated. The results did not differ by participant characteristics. Results to the RSA and results from the workshops that were co-hosted by RMs were reported in the plenary session and further discussed. The workshops, the RSA and the whole CPD conference raised awareness among health professionals about stigmatizing attitudes and epistemic inequity in actual service provision.

**Conclusion:**

RMs could be invited to actively participate and attend advisory boards and management meetings more frequently and on a more regular basis for ongoing quality improvement towards better access to recovery-oriented practices. This CPD conference has shown the acceptability and feasibility of including RMs as trainers for better recognition of the epistemic value of the experiential knowledge of recovery. They can help health professionals to recognize and better appreciate service users as knowers and potential contributors to knowledge.

## 1. Introduction

Mortality due to physical illness is 70% higher among mental health service users compared to the general population [1]. Responding to the needs of this disadvantaged group with high medical requirements is challenging for public health and primary care teams [2],[3] because many interrelated factors contribute to this poor health. The explanations generally point to individual lifestyle factors such as smoking, alcohol, physical inactivity, and a high body mass index. Serious mental illnesses, such as schizophrenia, are not, per se, life-threatening diseases. However, people with schizophrenia have a life expectancy reduced by almost 20 years compared to the general population [4]. These people die much younger, yet from the same complications of chronic physical illnesses that affect the rest of the population, such as respiratory diseases, cardiovascular diseases, or cancer, for example [5]. In their meta-analysis of studies of mortality and Major Psychiatric Disorders in 29 countries, Walker, McGee and Druss showed that the risk of premature death in people with psychoses, for example was 2.5 times that of the general population and that the median years of potential life lost was 10 years [6]. Possible causes for this disparity include: delays in preventive health examinations, delays in detecting problems leading to more advanced disease at the time of diagnosis, and delays in the deployment of vital treatments when the diagnosis is made.

From a public health perspective, the causes of diseases are: genetics, the social and physical environment, the lifestyle, and finally the health services received (or not received). There is a biological or genetic condition inherent in serious mental illness (SMI: schizophrenia, schizotypal disorder, and delusional disorder, as defined in Chapter 5 of the International Classification of Diseases), sometimes complicated by the effects of antipsychotic medication in metabolic terms. But moreover, a problem of communication and mutual trust between patients and health professionals for this vulnerable population further limits their access to care because these patients are often presumed to be irrelevant, unreliable, confused, or otherwise lacking in credibility. Studies have shown that even when treated in general practice, patients with schizophrenia are indeed less likely to receive thorough medical exams. Despite the best intentions, physicians often do not believe what psychiatric patients tell them. Such patients thus face additional barriers to getting their opinions heard on care and expected outcomes, whether in primary care contexts or through programmes supposedly designed with the intent of enabling their participation. Several negative consequences of this exclusion are well documented for this population, including poorer health outcomes and reduced life expectancy [7].

### 1.1. Epistemic inequity and diagnostic overshadowing

Doctors have great skills to search the body in a critical and systematic way to arrive at a diagnosis. However, this critical view of the diagnosis can be affected by other factors that combine into a phenomenon known as *diagnostic overshadowing* [8],[9]. The term was introduced in 1982 by Heiss, Levithan and Szyszko [10] to refer to this tendency for clinicians to attribute symptoms or behaviors of a mentally or intellectually challenged person to their underlying cognitive deficits and hence to under-diagnose the presence of co-morbidities, resulting in more advanced pathologies when they become evident. Among the consequences is that patient interpretations are not sought nor acknowledged as credible. They are thus undermined in their capacity as knowers and possible contributors to the effort to reach a proper understanding, diagnosis, and treatment. This diagnostic overshadowing phenomenon is amplified when people arrive with a long list of drugs including psychotropic medications, extensive medical history, and frequent visits to medical services or to the Emergency Room [11].

There is a need for health professionals to be trained to listen carefully to what psychiatric patients are telling them and to engage with them in collaborative decision-making, in order to allow psychiatric patients to have a greater role in their care and to overcome the risk of epistemic inequity. Indeed, Crichton, Carel, and Kidd [12] argue that the psychiatric patients' emotions are often taken by health professionals to have a detrimental effect on the patients' thinking, distorting the accounts they give of their condition. Such stereotypes, they say, include viewing mentally ill persons as cognitively impaired or emotionally compromised, owing either to their somatic condition or to their psychological reactions to it, or as existentially unstable, gripped by anxieties such that they “cannot think straight”. These people are particularly vulnerable to this type of inequity as a consequence of deeply embedded social stigma resulting in a priori assumptions of irrationality and unreliability such that their experiential knowledge is often discounted or downgraded [13]. It is important to acknowledge this epistemic inequity because of the persistent negative stereotypes that affect psychiatric patients in particular that lead to a credibility deficit. Health professionals should accept what these people say as true unless there is good reason not to.

### 1.2. Recovering epistemic equity

To address the epistemic inequity that is behind the diagnostic overshadowing phenomenon, a continuing professional development (CPD) conference has been organized, as reported here. This CPD activity has directly exposed health professionals to service users as knowers, more precisely to Recovery Mentors who can draw attention on recovery-oriented quality improvement and communicational barriers to access to health care and health professionals. Indeed, the concept of mental health recovery has decisively gained traction throughout the world.

The recovery paradigm refers to living a satisfying, hopeful, and contributing life, even when a person may still be experiencing ongoing symptoms. Recovery principles, including hope, dignity, self-determination, and responsibility, can be adapted to the full range of psychiatric disorders, and to the realities of different life stages. In the case of enduring psychiatric disorders, the information about such conditions need not be limited to the nature or etiology of the underlying pathology, but rather about how to live as satisfactorily and as independently a life as possible in spite of the persistence of these conditions while continuing to strive to reach one's full potential. The experience of living in recovery without necessarily being cured is thus particularly useful for sharing among peers who are coping, and/or have coped, with similar issues. The commonality is to the struggle and emotional pain that can accompany the feeling of loss and/or hopelessness due to a psychiatric condition, rather than in relation to a specific symptom or illness [14].

Then, Recovery Mentors (RMs) are persons who are further along in their recovery. They provide supportive services, for instance when hired to fill such a specialty position within Assertive Community Treatment programs [15]. When they share their own lived experience or when they comment and/or write notes on other specific patients' records for other members of the treatment team, RMs focus on health and recovery rather than illness and disability, and they draw attention to their capabilities and epistemic potential. They also act as community bridges who are accustomed to navigate the health and social benefits systems, and through a string of social determinants and organizations of different sectors [16]. They provide the mental health service users with whom they are in contact with—their mentees—a validation of their experience, and they facilitate their reclaiming their lives in the community, for example through self-advocacy. This relationship is founded on key principles of respect, shared responsibility, mutual agreement, and understanding another's situation empathically through the shared experience of emotional and psychological pain and overcoming adversity [17]. Indeed, patients served by case management teams with RMs have shown greater treatment engagement, more satisfaction with their life situation and finances, and fewer life problems than in comparison to case management alone [18],[19].

There now exist formal university programs to train RMs, for them to perform this translator/interpret role either on complex multidisciplinary teams or in community organizations [20]. Once graduated, they can also provide guidance to mental health teams interested in becoming recovery-oriented and in learning from the lived experience of recovery. This latter type of learning, termed Continuing Professional Development (CPD), goes beyond the concept of basic medical education as it encompasses personal as well as professional development [21]. This paper reports on an accredited CPD conference where RMs acted as trainers to inform and sensitize, about recovery and about the contributive potential of service users as knowers, the health professionals of a large health institution in Canada.

## 2. Material and methods

In March 2018, a conference was held in one of the *Integrated University Health and Social Services Centers* (IUHSSC) of the province of Quebec, Canada. The IUHSSC organization is the gateway to the public service system where the Quebec population can turn in case of health problems and/or psychosocial problems, including mental illnesses. Due to their university affiliation, IUSHSSCs contribute to academic training as well as to the development and dissemination of scientific knowledge. With regard to mental health, the Ministry of Health and Social Services of the province of Quebec expects the constituents of its network to integrate RMs into their treatment teams [22], as recommended by practice guidelines for the implementation of the recovery paradigm [23]. To prepare for the arrival of RMs in the teams of one of these IUHSSCs, a CPD conference was organized. A number of RMs co-hosted this collective learning and scientific event. Upon completion of their evaluation form at the end of the day, health professionals received a certificate for 5 hours of CPD, as recognized by the *Royal College of Physicians and Surgeons of Canada* and other professional associations like *Ordre des travailleurs sociaux du Québec* (Quebec College of Social Workers). At least 25% of the time had to be dedicated to interactive activities for this to be an accredited CPD conference, either between participants to self-reflect on their practice, or with the presenters who were RMs in this particular case. The Recovery Self-Assessment (RSA) was used to trigger this reflexivity.

### 2.1. Measure: Recovery Self-Assessment (RSA)

The RSA tool was designed to gauge the degree to which programs implement recovery-oriented practices [24]. It is a self-reflective tool designed to identify strengths and target areas of improvement as agencies and systems strive to offer recovery-oriented care. The RSA contains concrete, operational items to help program staff, persons in recovery, and significant others to identify practices in their agency that facilitate or impede recovery. The RSA has five factors: diversity of treatment options (Cronbach's alpha = 0.86), consumer involvement and recovery education (Cronbach's alpha 0.86), life goals vs. symptom management (Cronbach's alpha = 0.76), rights and respect (Cronbach's alpha = 0.71), and individually-tailored services (Cronbach's alpha = 0.75). The Cronbach's alpha for the whole scale is of 0.94 [25].

The RSA is among the most widely used rating scales to facilitate reflection on the strengths and limitations of services within a recovery framework [26]. It is intended for use with individuals who receive and/or provide services in inpatient settings, outpatient settings, peer-run programs, residential programs, and social programs. The RSA questionnaire has versions for administrators, service providers, family members/key supports, and service users. It is the version for service providers that was used for CPD purposes. RSA items cover five domains: *Life goals versus symptom management*; *Consumer involvement and recovery education*; *Diversity of treatment options*; *Rights and respect*; and *Individually tailored services*. The RSA allows for a generation of a total mean score, domain means, and for the comparison of stakeholder perspectives [27]. The RSA has undergone varying degrees of psychometric testing including examination of internal consistency, test– retest reliability, content, convergent, and discriminant validity [28],[29]. It can thus be used as a reliable and valid measure of recovery orientation that can be used to assess a variety of mental health programs The scale showed reliability in prototype [30] and underwent appropriate processes of item development (drawing on stakeholders' input) and testing (using techniques such as concept mapping, principal components analysis and factor analysis), and has been published in peer-reviewed journals [31].

### 2.2. Participants

Clinicians: Among the 105 participants who registered, 88 completed the RSA and 57 clinicians completed their evaluation form to receive their CPD credits (Table 1, no missing data). They were all employees of the IUHSSC.

**Table 1.** Profile of participating clinicians (n = 57).

	N	%
Sex		
Male	15	23%
Female	42	68%
Age group		
20–29	11	19%
30–39	21	35%
40–49	13	23%
50–59	12	21%
Profession		
Planning, programming and research agent	4	7%
Occupational therapist	6	11%
Nurse	18	30%
Psychologist	5	9%
Social worker	21	33%
Other	3	5%
Years of practice		
Less than 5	12	21%
5 to 9	13	23%
10 to 19	23	40%
20 to 29	4	7%
30 to 39	5	9%

Recovery Mentors: Among the 9 RMs who were involved, 6 were female and 3 were male. They were trained by the Department of Psychiatry of the University of Montreal and recruited through their professional association, which started as a peer-run agency of service users who came together as a private non-profit organization to promote their experiential knowledge in science and public mental health debates [32].

### 2.3. The tracers method

Engaging people with lived experience extends beyond their participation as “subjects”. In fact, the recovery paradigm conceptualizes people with lived experience of mental health problems or illnesses as true experts by experience. Their engagement improves sensitivity and respect, for example through recognizing the importance of having a language that moves away from a problem-saturated view to a shared language about hope and possibility. To support the transformation process, professionals should be able to move beyond a role defined primarily by diagnosis and medication management [33]. To do so, they need to acquire the skills and understanding to develop trusting and nurturing relationships with service users living with psychiatric disorders. RMs were thus paired with health professionals with the goals of improving the workshops participants' understanding of recovery and reducing negative stereotypes about people in recovery [34]. Participants were distributed into structured workshops for small group learning. Small group learning is an educational approach that allows participants to develop problem solving, interpersonal, presentational, and communicational skills that are difficult to develop in isolation, and that require feedback and interaction with others [35]. As suggested by Rudnick and Eastwood, to train medical staff to envision mental health service-users differently through their potential and capabilities rather than through their symptoms [36], during the workshops the RMs shared their own experience of recovery by focusing on transition points in the system in order to highlight areas of risk, inefficiency and redundancy. This approach to teaching emphasizes the importance of addressing attitudes in addition to knowledge and skills and it aligns with the *Canadian Medical Education Directives for Specialists* (CanMEDS) [37].

While co-learning methods include problem-based learning and simulations, the tracers method aims to highlight potential trajectories that characterize a system [38]. This method allows the identification and description of organizational processes and relational networks over time and goes beyond the description of a process from a pre-built model. It thus becomes possible to document a variety of issues experienced in the field, but also to find possible solutions to them. This method aims at understanding the dynamics in which the subject evolves and from his or her own perspective. For health organizations, the patient-tracer method is used to improve the quality of care and the patient experience. In particular, it allows for the retrospective analysis of the quality and safety of a patient's management throughout the institution's journey, as well as inter-professional and interdisciplinary interfaces and collaboration in order to identify and implement improvements [39]. We applied this method to the multidimensional post-acute care recovery trajectory through storytelling that RMs can perform with their own recovery narrative, for this specific purpose of empathic understanding. The tracer method specifically aimed to identify self-determined processes of recovery in order to unveil the arrangement of mechanisms and procedures that mark this process. The contribution of RMs was to act as pathfinders in order to illustrate the succession of actors, contexts of action, conditions of action and the processes involved in this multidimensional recovery trajectory. In preparation for the interactive workshops hosted by RMs, participants were asked to complete the Recovery Self-Assessment (RSA). The results (1) from the RSA and (2) from the workshops were discussed in the plenary session, which was also audio-taped. A note-taking canvas was used to structure observations beforehand and to capture the interaction among workshops participants, and then to report convergent or divergent point of views to the final plenary session where these reports where compared for recommendations synthesis. Workshop facilitators were trained to observe the same areas of interest and to report to the plenary session.

## 3. Results

*Workshops*: Reports from the concurrent workshops were compared by teams of trainers. It was commonly noticed that the work of RMs is now a growing profession, which was found to be encouraging for the recognition of the experiential knowledge. A better awareness of some stigmatizing behaviors and beliefs at play in the actual service provision, such as the use of a somewhat condescending and fatalistic vocabulary, was also reported. The narrative of the RMs was suggested as a lever of change to further tackle stigma and learn about the recovery potential of the service users in general.

Self-stigma was also discussed. The key role of RMs was underlined to reassure and promote the involvement of the service user in his/her care and therapeutic alliance with the treatment team. RMs' role is to empower service users to overcome self-stigma that reinforce cognitive and social challenges associated with stigma against psychiatric illnesses and which dilutes the potential efficacy of the therapeutic alliance [40]. Addressing stigma and self-stigma were presented as a prerequisite for person-centeredness with a focus on the service user's life project and post-hospital trajectory rather than on the service user as a “walking illness” (as noted and reported).

The teachings from RMs were globally deemed helpful in understanding the impact of the recovery approach, with stability and continuity of support appearing to be of a central importance in the long term given that recovery is a non-linear process with normal occasional setbacks. It was suggested that the specific expertise of RMs transcends the traditional categories of thought and bridges organizational silos [41]. Nevertheless, the magnitude of cultural change required to make room for RMs and integrate recovery principles into daily practice was not to be underestimated. For instance, it was suggested to be more acutely aware of the involuntary and somewhat automatic language that can be disrespectful in team meetings. With a new vocabulary based on the ordinary language might come a whole different mindset that should focus on strengths in order to avoid the pre-labeling by clinical staff of some patients as frustrating or hopeless. Health providers were also wondering if RMs could rely on a professional network for support and for their own continuing professional development.

*The Recovery Self-Assessment*: Means and standard deviation for each item of the RSA questionnaire were calculated with the IBM Statistical Package for the Social Sciences (SPSS Statistics 24). Based on the average inter-item correlation, a reliability test confirmed an excellent internal consistency for the French RSA scale, with a Cronbach's Alpha of 0.9. The results did not differ by participant characteristics. Table 2 shows the results of the RSA. These results were presented just after the workshops at the beginning of the final plenary session and thus before the presentations of the reports from the workshops.

## 4. Discussion

The Actor-Network Theory (ANT) was developed in the field of science and technology studies during the 1980s [42]. ANT ascribes equal agency to people and to different types of experience or knowledge, and this implies that no type of knowledge is *a priori* superior to another. Inherent to ANT is a move away from the idea that innovation impacts on humans as an external force, to the view that innovation emerges from interests (e.g. professional) and that it therefore has the potential to shape social-professional interactions [43]. ANT has been used to study patient experience [44], and for exploring changing power relationships in relation to healthcare reforms [45]. It is commonly used to investigate and theorise about how networks come into being, how actors are enrolled into a network, and how parts of a network come to form a whole network [46]. An overarching aim of this CPD day was to bring parts of this IUHSSC to form a whole and single recovery-oriented mental healthcare network with the introduction of a new actor in this network, namely RMs as holders of a different type of knowledge. Indeed, ANT assumes that if any actor, irrespective of its position, is removed from or added to a network, then the functioning of the whole network is affected [47]. This system of mutual influence suggests that actors (for instance, mental healthcare providers and RMs in our case), act in the way they do and are able to produce effects only through their interactions with others. We thus organized workshops as a means to put such providers and RMs in interaction, as discussed in this section.

**Table 2.** Results of the French Recovery Self-Assessment for providers (n = 88).

RSA item	Mean	Standard deviation
1. Staff make a concerted effort to welcome people in recovery and help them to feel comfortable in this program	4.0	1.0
2. This program/agency offers an inviting and dignified physical environment	3.0	1.2
3. Staff encourage program participants to have hope and high expectations for their recovery	3.8	0.8
4. Program participants can change their clinician or case manager if they wish	2.2	1.1
5. Program participants can easily access their treatment records if they wish	2.9	1.2
6. Staff do not use threats, bribes, or other forms of pressure to influence the behavior of program participants	4.0	0.9
7. Staff believe in the ability of program participants to recover	4.2	0.7
8. Staff believe that program participants have the ability to manage their own symptoms	3.8	0.8
9. Staff believe that program participants can make their own life choices regarding things such as where to live, when to work, whom to be friends with, etc	3.9	0.9
10. Staff listen to and respect the decisions that program participants make about their treatment and care	3.8	0.7
11. Staff regularly ask program participants about their interests and the things they would like to do in the community	3.9	0.8
12. Staff encourage program participants to take risks and try new things	4.0	0.7
13. This program offers specific services that fit each participant's unique culture and life experiences	3.0	0.9
14. Staff offer participants opportunities to discuss their spiritual needs and interests when they wish	3.3	1.1
15. Staff offer participants opportunities to discuss their sexual needs and interests when they wish	3.1	1.2
16. Staff help program participants to develop and plan for life goals beyond managing symptoms or staying stable	4.1	0.7
17. Staff routinely assist program participants with getting jobs	4.1	0.9
18. Staff actively help program participants to get involved in non-mental health/addiction related activities, such as church groups, adult education, sports, or hobbies	3.9	1.0
19. Staff work hard to help program participants to include people who are important to them in their recovery/treatment planning	3.5	1.0
20. Staff actively introduce program participants to persons in recovery who can serve as role models or mentors	2.3	1.1
21. Staff actively connect program participants with self-help, peer support, or consumer advocacy groups and programs	3.5	1.0
22. Staff actively help people find ways to give back to their community	3.5	1.1
23. People in recovery are encouraged to help staff with the development of new groups, programs, or services	2.7	1.3
24. People in recovery are encouraged to be involved in the evaluation of this agency's programs, services, and service providers	2.5	1.1
25. People in recovery are encouraged to attend agency advisory boards and management meetings	2.2	1.2
26. Staff talk with program participants about what it takes to complete or exit the program	3.4	1.2
27. Progress made towards an individual's own personal goals is tracked regularly	3.9	0.8
28. The primary role of agency staff is to assist a person with fulfilling his/her own goals and aspirations	4.1	0.8
29. Persons in recovery are involved with facilitating staff trainings and education at this program	2.4	1.3
30. Staff at this program regularly attend trainings on cultural competency	2.2	1.2
31. Staff are knowledgeable about special interest groups and activities in the community	3.5	1.0
32. Agency staff are diverse in terms of culture, ethnicity, lifestyle, and interests.	3.4	1.2

During the final plenary session, results from the workshops and from the RSA were crossed and combined. This exercise led to the conclusion that most of the IUHSSC staff do believe in the ability of service users to be in recovery, but not all of them. They also expressed a need to be better equipped with specific recovery tools and training because otherwise, recovery might not happen spontaneously by itself simply because there would now be RMs in the teams. They were nevertheless impressed and even moved by the RMs, with whom they would have liked to interact even more to learn from them. They were welcoming towards RMs and said that they were looking forward to working with them. As of July 2019, 6 RMs are now integrated in this IUHSSC through contracts with their private non-profit organization.

It was also recommended that RMs should actively attend the advisory boards and institutional management meetings for continuing and ongoing organizational quality improvement. They should do so more frequently and on a regular basis for a lasting change of culture and attitudes, a transformation that might be evaluated with a formal protocol for future research (e.g., pre-post design). Some suggested that this should have been done long ago anyway.

The grouping of RMs within their own professional association might help to promote their formal recognition as a new profession for stronger integration into multidisciplinary teams where everyone else is a member of his/her professional association. The risk of losing their identity and spontaneity if they were becoming “too professional” was raised. A collectivistic approach would allow them to support each other and to gain recognition from the professional identity they would give themselves first, if the goal is to admit them fully while preserving their difference and protect diversity. In their evaluation form at the end of the day, some professionals did express reluctance with the basic assumption that people with lived experience can and should teach them how to do their jobs better, but this was not reported nor expressed during the plenary session. In any case it was not about telling them what to do or not, but about realizing that RMs and service users can be valuable contributors to broadening their understanding.

Indeed, as suggested by Miller Tate (2019) the term “contributory injustice” marks out a typical feature of the epistemic inequity where the marginalized are unable to contribute equally to the collective understanding of their experiences because their contributions are systematically dismissed [48]. This closely relates to Fricker's concept of hermeneutical injustice, but while hermeneutical injustice refers to cases where both the marginalized and dominant groups share a lack of epistemic resources needed to express or understand the former's experiences, contributory injustice picks out cases where relevant resources have been developed and used by the marginalized group, but not taken up by the dominant group [49]. Addressing epistemic inequity and stigma must be undertaken on every level—from academic leaders in the health profession at large, to psychiatric educators, and to undergraduate and graduate trainees for genuine uptake of the experiential knowledge [50]. On one hand, involving RMs as employees of mental health services can lead to service users having greater satisfaction with personal circumstances and less hospitalisation. On the other hand, as reported by Simpson and House, service providers who are trained by RMs can develop more positive attitudes toward service users. Clients admitted being less satisfied with services when interviewed by users [51].

## 5. Limitations

It is not possible at this point to attribute separate or specific effects to either the use of the RSA or to the participation in interactive workshops to stimulate reflexivity among mental healthcare providers. More research is needed to isolate such possible effects among them, between different categories of professionals or in terms of years of practice, for example. Now that the acceptability and feasibility of such an approach is documented, it would be possible and relevant to more accurately and more specifically evaluate its effectiveness with a pre-post research design to verify if providers do become more knowledgeable, and to what extent, about recovery and the RMs contributive potential.

## 6. Conclusion

This CPD conference has shown the acceptability and feasibility of including RMs as trainers for better recognition of the epistemic value of their own experiential knowledge and more broadly that of persons in recovery. RMs can help and train health providers to recognize and better value service users as knowers and potential contributors for enlarged understanding. This is an appropriate way of promoting epistemic equity and thus access to genuinely recovery-oriented services.
